# Bringing Light into Darkness—Comparison of Different Personal Dosimeters for Assessment of Solar Ultraviolet Exposure

**DOI:** 10.3390/ijerph18179071

**Published:** 2021-08-27

**Authors:** Claudine Strehl, Timo Heepenstrick, Peter Knuschke, Marc Wittlich

**Affiliations:** 1 Department Ergonomics: Physical Environmental Factors, Institute for Occupational Safety and Health of the German Social Accident Insurance, D-53757 Sankt Augustin, Germany; timo.heepenstrick@dguv.de; 2 Department of Dermatology, Faculty of Medicine, Technische Universitaet Dresden, D-01307 Dresden, Germany; knuschke@rcs.urz.tu-dresden.de; 3 Department Accident Prevention: Digitalisation–Technologies, Institute for Occupational Safety and Health of the German Social Accident Insurance, D-53757 Sankt Augustin, Germany; marc.wittlich@dguv.de

**Keywords:** personal dosimetry, UV radiation, intercalibration

## Abstract

(1) Measuring personal exposure to solar ultraviolet radiation (UVR) poses a major challenges for researchers. Often, the study design determines the measuring devices that can be used, be it the duration of measurements or size restrictions on different body parts. It is therefore of great importance that measuring devices produce comparable results despite technical differences and modes of operation. Particularly when measurement results from different studies dealing with personal UV exposure are to be compared with each other, the need for intercomparability and intercalibration factors between different measurement systems becomes significant. (2) Three commonly used dosimeter types—(polysulphone film (PSF), biological, and electronic dosimeters)—were selected to perform intercalibration measurements. They differ in measurement principle and sensitivity, measurement accuracy, and susceptibility to inaccuracies. The aim was to derive intercalibration factors for these dosimeter types. (3) While a calibration factor between PSF and electronic dosimeters of about 1.3 could be derived for direct irradiation of the dosimeters, this was not the case for larger angles of incidence of solar radiation with increasing fractions of diffuse irradiation. Electronic dosimeters show small standard deviation across all measurements. For biological dosimeters, no intercalibration factor could be found with respect to PSF and electronic dosimeters. In a use case, the relation between steady-state measurements and personal measurements was studied. On average, persons acquired only a small fraction of the ambient radiation.

## 1. Introduction

During the first decades of this century, skin cancer became a widespread disease [1,2]. Solar radiation accompanies us always and everywhere and therefore cannot be avoided completely. For this reason, efforts to discover the effects of UV radiation (UVR) on our cells have to be extended. Determination of the extent of personal UV exposure plays a key role in understanding the mechanisms of skin cancer development [3]. This is in addition to prevention activities by different stakeholders, legislation on preventive measures, or even limit values based on exposure measurements and the conclusions drawn from them. This makes it possible to determine when and where people are exposed the most and to develop tailor-made prevention strategies for both leisure time and occupational behavior [4].

Success in prevention relies on balanced measures of structural and behavior-centered prevention [5]. Structural prevention pursues the goal of exerting a positive influence on health by shaping living, working, and environmental conditions e.g., by means of statutory regulations, or with a focus on solar UVR urban planning measures or implementation of protective measures at the workplace. In contrast, behavior-centered prevention directly targets people to influence health-related behaviors like the use of sunscreen or tanning beds. All of these strategic measures can be substantiated with measurement data that can provide information on real personal UV exposure.

There are a number of different methods to measure personal exposure to UV doses [6,7]. All of these different measurement systems have their own characteristics that have to be taken into account when choosing one of them for personal dosimetry. The most commonly used dosimeters are probably polysulphone film (PSF) dosimeters, which are based on the change of optical absorbance of a thin polysulphone film by exposure to UVR [8,9,10,11,12]. Recently they have been replaced by electronic dosimeters which provide the possibility of gathering more in-depth details. Electronic dosimeters, which are able to directly measure UV doses, are provided in watch-like designs [13] or as data loggers [14,15], and can be attached to different parts of the body such as the chest or the limbs. A further type of actinic UV sensors used in personal UV dosimetry are biological dosimeters using the germicidal effects of UVR on spores to quantify UV exposure [16,17,18].

As PSF dosimeters only measure the cumulative amount of UV exposure during the measurement period, they do not allow temporal resolution of measurement data. Therefore, a validity check of the acquired measurement data, e.g., in terms of correct attachment to measurement sites, is not possible. Additionally, the PSF is insensitive to radiation with a wavelength greater than 340 nm due to its material properties [19,20]. This means that the UVA portion of the spectrum has to be extrapolated during a calibration process against the sun spectrum without knowledge of the real exposure—on the other hand, the actinic fraction of the UVA exposure is low and in this way the uncertainty too [11,21,22]. This applies also to biological dosimeters, where the uncertainty is aggravated by questions on the temperature stability of the germs. After usage, the active media of the dosimeters have to be discarded while the shell can be reused. Polysulphone film and biological dosimeters score with relatively low prices for mass measurements and mechanical robustness against shocks, vibration, and strong electromagnetic fields.

The clear advantage of electronic data logger dosimeters is the temporal resolution of measurement data. The first intercalibration measurements between several personal UV dosimeter types, available at that time were carried out in 2004 [23]. For this study, the intercalibration of personal UV dosimeters available in 2004 was performed on three clear sky days with defined noon solar elevations (60°, 42°, 30°) simultaneously to a double-monochromator spectroradiometer. The 2004 study included PSF, X2000-4 (Fa. Gigahertz-Optik, Türkenfeld, Germany), UVDAN datalogger dosimeters (Fa. ESYS Berlin, Germany), and biological UV dosimeters (VioSpore, Fa. Biosense Borheim, Germany). In a field trial intercalibration, six outdoor workers wore these four dosimeters as a 4-in-1 dosimeter at the workplace for five days. For the X2000-4 datalogger dosimeter, the intercalibration revealed that the type used as an actinic UV sensor at that time showed specimen scatterings with significant deviations from the spectral sensitivity to the erythema action spectrum, resulting in measurement deviations up to a factor of 2 in the course of a day. Based on this knowledge, the company developed a dual sensor system (UVA and UVB/C) with improved spectral sensor response (called X2010). The new system served as the basis for innovative extensions (see Section 2, Materials and Methods). In light of this, the formerly-derived intercalibration results cannot be used for this new generation of UV data logger dosimeters.

Beside the advantage of the time-resolved measurements of the UV data logger dosimeters, an increase in performance is possible by using additional features (software or sensors). By using an integrated acceleration sensor, it is possible to ensure the quality of acquired data, e.g., by checking if the dosimeters were worn correctly during the measurement period. Though electronic dosimeters are more expensive in acquisition and maintenance, they can be reused for years. All of these characteristics make it hard to draw direct comparisons between measurement data on personal UV exposure acquired with different measurement systems.

This paper focuses on a comparison of the different measurement methods with the goal of the elaboration of the intercalibration factors between the measurement systems. The main perspectives are to determine to what extent measurement data acquired with different measurement systems is intercomparable, and to establish the limiting factors of intercalibration. Furthermore, this paper provides information which may assist decisions on which method is most suitable for a specific measurement task. In the context of comparison between steady-state and personal UV exposure acquired with electronic dosimeters, a use case for personal UV monitoring will be presented.

## 2. Materials and Methods

### 2.1. Dosimeter Types and Principle of Measurement

Polysulphone film dosimeters are based on the change of optical absorbance of the PSF by exposure to UV radiation [8]. The spectral sensitivity of this effect is similar to certain action spectra, e.g., the erythemal weighting function s_er._ The erythemal weighting function resembles the UV erythema reaction of the skin, and is therefore used to describe the spectral dependence of this effect [20]. At 330 nm, the optical absorbance of the film is proportional to exposure. Optical properties of the PSF are determined before and after exposure in order to calculate the difference between both values as a measure of exposure [21]. A correction factor yields the biological effectiveness of UVR [11]. PSF dosimeters are usually provided with reusable housings. Metallic meshes can be used as neutral filters to extend the measuring range by attenuating incoming radiation. The measurement range of PSF dosimeters ranges from 0.5 kJ/m^2^ to 300 kJ/m^2^ (@295 nm) [11]. The PSF dosimeters used in this work were purchased and evaluated by the Technische Universitaet Dresden, Department of Dermatology, Section: Experimental Photobiology, Germany.

Spore and biofilm UV dosimeters are based on measuring the survival rate of *Bacillus subtilis* spores exposed to UVR [24,25]. For this purpose, spores are immobilized on a carrier. The carrier is fixed in a housing that can be mounted to the measurement site. In order to evaluate exposure of the dosimeters, it is necessary to compare them with dosimeters exposed to a reference exposure in the laboratory. Subsequently, the spores are incubated, the samples are stained, and the colony-forming ability of the spores as a measure of the UV dose is determined using an image processing system [26,27,28,29]. The spectral response curve of these spores combined with upstreamed filter foils is very similar to the s_er_ function [16]. Two different types of spore dosimeters were chosen for our measurements: the VioSpor blue line type II with a measurement range of 1 SED to 55 SED (Standard Erythema Dose, 100 J/m^2^ erythemal weighted radiation) and VioSpor blue line type III dosimeters 1.5 SED to 90 SED from BioSense (Bornheim, Germany) [30]. After measurement, the spore dosimeters were processed and evaluated by the manufacturer according to the procedure described above.

The electronic dosimeters used in this work (X2012-10 version 1 (V1) and version 3 (V3), from Gigahertz Optik GmbH, Türkenfeld, Germany) detect and measure erythema-effective UVR in time steps of 1 s in two channels, UVA and UV B/C, respectively. The s_er_ weighting function is physically realized by optical filter packages within the scatter discs on the diodes. V1 and V3 share the same technology for the optical system. The dosimeters come with software and algorithms for data recalibration and evaluation.

### 2.2. Measurement Setup

A sun-tracking device was used to achieve the desired intercalibration measurement for the different types of dosimeter. With this device, it was possible to maintain a constant angle between the dosimeters and the sun throughout the day. For each measurement, the following dosimeters were mounted onto the device:

- 4 units of X2012-10 V1.
- 4 units of X2012-10 V3.
- 5 PSF dosimeters.
- 5 units of VioSpor blue line Type III.
- 2 units of VioSpor blue line Type II* (* These dosimeters were only used for measurements 1–4).

**Figure 1:**
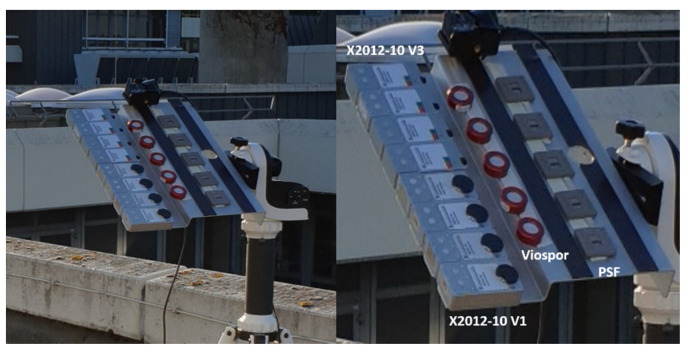
All dosimeters mounted on the sun-tracking device on the roof of the Institute for Occupational Safety and Health, Sankt Augustin, Germany (left-hand side: electronic dosimeters X2012-10 V3 (above) and V1 (below); in the center: VioSpor blue line type III; right-hand side: Polysulphone film (PSF) dosimeters). Shading caused by buildings or trees was avoided.

Polysulphone film and spore dosimeters were single-use only, while electronic dosimeters were read out after measurement. Afterwards, the internal memory was erased and the dosimeters could be reused for subsequent measurements. A picture of the setup is shown in Figure 1. In total, eight different measurement scenarios were chosen, varying in time of day, meteorological conditions and angle towards the sun. For each measurement scenario, the incident angle of solar radiation was kept constant over the exposure period by the sun-tracking device. The measurements took place in May, June, and September 2020 in Sankt Augustin (50.8° N, 7.2° E), Germany. Stable, dry weather conditions were a prerequisite for performing the measurements throughout the entire measurement period, as most of the dosimeters are not water resistant. The measurement parameters for the different scenarios are shown in Table 1.

### 2.3. Use Case GENESIS-UV

Direct linkage between personal behavior and personal UV dosimetry may become crucial for understanding and predicting UV exposure in the future using steady-state UV measuring networks or satellite prediction data [31]. For this purpose, we used a mobile weather station (MAWS201, Vaisala, Hamburg, Germany) equipped with three identical pyranometers to assess global, ambient, and reflected UVR, respectively. At a local company event, we equipped 39 test persons with X2012-10 dosimeters. The MAWS and additional static dosimeters were run in parallel. The event took place in July 2018, on a cloudless day just a few weeks after summer solstice. As pyranometers are considered to be the gold standard in radiometry of optical radiation, a comparison of both steady-state and personal UV measurements provides a good and reliable statement on the quality of dosimetric measurements. The MAWS201 and its devices came calibrated to international standards. Nevertheless, intercomparison measurements with the German Meteorological Service (Deutscher Wetterdienst, DWD) were conducted to ensure highest comparability to publicly available data.

Wearable dosimeters of type X2012-10 were developed as part of the GENESIS-UV measurement system [32]. This system is used for decentralized long-term measurement of UV exposure of people assessing occupational UV dose. The measurement system consists of 300 independent units. The core of each unit is represented by an X2012-10 dosimeter. In order to allow long-term mapping of personal UV exposure, the dosimeters are combined with a data client available on a supplementary tablet PC. By means of the data client, which is a special software installed on the tablet PC, measurement data was automatically extracted from the dosimeters by periodically connecting them to the tablet PC after one or more days of measurement. This data was subsequently stored on a server, located at the research center. From there, the data could be further analyzed and evaluated. 

The same experimental setup with MAWS and X2012-10 was also used during a low ozone event in April 2017 in Didcot, England [33].

## 3. Results

### 3.1. Intercalibration Measurements

**Figure 2:**
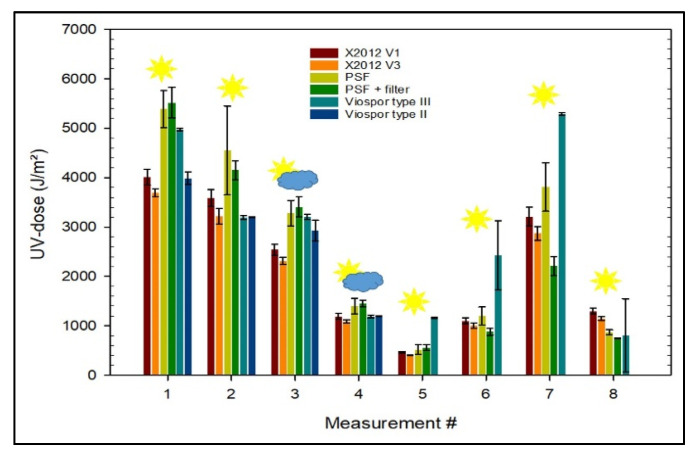
Chart of measurement data subdivided into measurement scenarios and dosimeter types. Incident angle of solar radiation: 0° for measurements number 1, 3–5; 30° for measurement number 2; 110° for measurement number 6; 60° for measurement number 7; 85° for measurement number 8.

The mean values of the collected UV doses for all measurement series, including their standard deviation, are shown in Table 2. A graphical representation of measurement data is depicted in Figure 2. It became apparent that data from one of the type X2012-10 V3 electronic dosimeters involved was corrupted due to a technical failure. Consequently, measurement data acquired with this device was excluded from the analysis.

When directed towards the sun, the three different dosimeter systems corresponded intrinsically to each other, i.e., V1 to V3, PSF to PSF plus Filter, VioSpor Type II to VioSpor Type III. The only exception are VioSpor dosimeters at measurement number 1. Values from generations V1 and V3 of the electronic dosimeters varied in an order of 10% throughout all measurements, with only small standard deviation within the sets of identical dosimeters. Only spore dosimeters showed comparably low standard deviation (except for measurements number 6 and number 8), while PSF dosimeters scattered more clearly. Polysulphone film dosimeters generally provided higher dose values compared to electronic dosimeters. Differences ranged from 20% to 30% for these two dosimeter types. With regard to the spore dosimeters, there was a more heterogeneous situation: compared to the other dosimeter types, no clear tendency of results (factor between spore dosimeters and PSF or electronic dosimeters) could be determined, although results themselves showed only a small standard deviation between related measurements. Possibly the reason for this is a higher natural variability of the germicidal effects in terms of the reaction of biological material to UVR.

The variations in measurement data become larger when scattered radiation, i.e., from the celestial hemisphere, became dominant and direct radiation was shielded. Essentially, units of the same type varied only slightly. Overall, the measured values from the electronic and PSF dosimeters were more comparable, but the spore dosimeters in particular clearly stand out. With the exception of measurement number 8, the spore dosimeters in particular indicated a significantly higher irradiation, combined with a high standard deviation of the individual measured values. In contrast with measurements of direct solar irradiation, no clear tendency between the measurements with the electronic dosimeters and the PSF dosimeters can be determined. The deviations are in the order of 10%, with the exception of the last measurement (40%) at a grazing incident angle of 85°. In this case, the measurements between PSF with and without filters differed significantly, because of the limited cosine response of the wire mesh filters. Shadowing of the PSF in the dosimeter badge due to frames and components lowers the incoming radiation substantially [23,34].

Considering all measurements and dosimeter types, the electronic dosimeters showed the lowest standard deviation, which is a clear sign of good intrinsic response behavior and calibration.

### 3.2. Comparison of Steady-State to Personal UV Exposure

**Figure 3:**
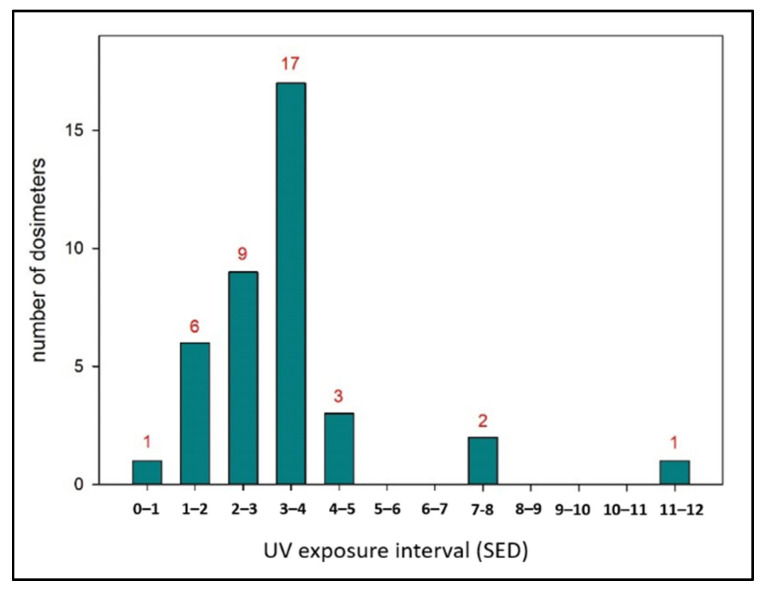
Histogram of individual UV exposure in units of Standard Erythema Dose (SED) measured within the framework of a company party by electronic dosimeters of type X2012-10. Red numbers indicate the number of dosimeters (results) for each exposure interval. Number of participants: 39; mean value: 3.4 SED; standard deviation: 1.9 SED; maximum value 11.1 SED; minimum value: 0.6 SED; global irradiation: 25.6 SED.

Within the framework of a single-day company event in mid-July, a group of 39 people were equipped with dosimeters at the same time. The meteorological station and static X2012-10 dosimeters were set up at the same location. The distribution of the individual UV exposure, subdivided into intervals of SED, is shown in Figure 3.

The statically mounted dosimeters showed good agreement with the pyranometers of the weather station during several measurement sessions at various locations and could therefore serve as a standard. Three static dosimeters were operated: for direct radiation with a total irradiation of 25.6 SED, for radiation reflected from the ground with a total irradiation of 1.0 SED, and for ambient radiation (i.e., without the solar disk, realized by placing the dosimeter underneath a shade ring) with a total irradiation of 19.3 SED, in each case related to the measurement period in which the test persons also wore the dosimeter.

From the individual UV exposure of the test persons, a mean value of 3.4 SED with a standard deviation of 1.9 SED was derived. Three particularly high exposure values greater than 7 SED occurred, which turned out to be correct measurements on closer examination of the values and dosimeters; there were no technical defect, and the method of carrying was within the expected variations.

## 4. Discussion

Overall, our measurements showed that there is a systematic difference between electronic dosimeters and PSF dosimeters, especially at almost perpendicular incidence on the detector surface. This can be described by a factor of 1.3, by which the PSF dosimeters overestimate exposure. Especially at higher angles of incidence (>30°), however, this factor no longer becomes apparent. In addition, no clear tendency can be determined between the two dosimeter types at large angles. This is particularly important if the dosimeters are to be attached to different parts of the body in practice and then have different orientations towards the radiation source. The sensor material, 26 µm thick PSF without a dosimeter badge, is characterized by a good cosine response [34]. Housed in the dosimeter badge, it is recessed for mechanical protection. This badge design limits its angle of view to about ±70° to the perpendicular measurement direction. Since a cosine response is also postulated for the skin [35], such non-detected UV rays (>±70°), which correspond to striking the skin flat, contribute only slightly to the total exposure coming from the half-space. Nevertheless, in comparison to UV measuring devices matching the cosine response more accurately, these amounts contribute to deviations, which can be seen in the intercalibration to precision instruments [36,37]. If 26 µm PSFs are to be used for global radiation survey measurements of up to 2-week periods, this effect can be achieved by additional grey filters to extend the measurement range, e.g., by metal gauze [38]. The double layer of metal gauze used in one of the two measurement fields of the investigated PSF badges, limits the angle of view to about ±45° to the perpendicular measurement direction. This lack of the viewing angles was taken into account by a spectroradiometrically-controlled calibration from a homogeneously radiating half-space, constant in time. These conditions are not valid in cases of survey measurements of the global radiation over one or more diurnal cycles. In case of low solar elevation, a PSF field with the double layer of metal gauze underestimates the direct radiation component of the global radiation, and the same is valid for other nonhomogeneous UVR fields. Therefore, the above-mentioned measurement setup (PSF plus 2 layers of wire mesh) is suitable only for rough estimation in personal dosimetric long-term measurements.

The same effect can also be seen in the biological spore dosimeters. Here, too, the highest deviations from the other dosimeters and intrinsic fluctuations occur at larger angles of incidence and ultimately smaller visible radiating areas. At direct irradiation and small angles, the spore dosimeters do not show any direct tendency and cannot be correlated with either electronic or PSF dosimeters by any factor. The measured values vary in the interval between the other dosimeter types.

The measurement of optical radiation is fundamentally subject to uncertainties [37,39]. Therefore, a certain deviation between measurement methods is to be expected from the outset. Nevertheless, calibration (e.g., following DIN 5031-11 [40], a German standard which defines the characteristics of radiometers used for measurement of actinic radiation), which is traceable to national standards given for each measurement method should lead to similar measured values [41]. In the factory, calibration is usually only carried out with vertically incident radiation on the detector. The cosine response of the detector, which defines the ability to detect incoming radiation of larger incident angles e.g., scattered radiation, is not always checked.

Recently, Zoelzer and Bauer [42] evaluated different dosimeter types with regard to their spectral responsivity. They compared them to different action spectra representing different biological effects, such as the CIE s_er_ erythema weighting function, to verify their ability to model this biological effect over the course of the year and day. Investigated dosimeters included PSF and biological dosimeters. The spectral responsivities were derived from published work. It could be shown that responsivities of the biological dosimeters are very close to the CIE erythema function. Thus, the resulting measured doses do not need any corrections with regard to annual and diurnal variation of the solar zenith angle. For PSF dosimeters, the response curve is slightly different compared to s_er_, especially in the wavelength range between 300 nm and 340 nm. This results in a need of diurnal and annual correction factors. The calibration factors can vary throughout the course of day. This has to be taken into account when evaluating the dosimeters. In comparison to this, in electronic dosimeters it is possible to implement a software application that immediately corrects every data point with respect to the current solar zenith angle.

It can be stated from our findings that dosimeters of different types can be compared with each other to a limited extent. The deviations of the measured values from each other were within a range of 30%, especially with a high proportion of radiation coming from a wide angular range of the sky. Accordingly, all dosimeter types are suitable depending on the intended use. Electronic dosimeters in particular are suitable for the detailed analysis of UVR exposure, as they demonstrate high data resolution and reproducibility, although the high price is a disadvantage. This is what makes them particularly suitable for use in long-term measurements. In contrast, PSF and spore dosimeters are suitable to serve measurements in high numbers. Especially if the temporal resolution of the irradiation does not play a major role, these dosimeters are robust and fast in use. Another important factor is the sustainability of electronic dosimeters. Whereas PSF and spore dosimeters can only be used once without the possibility of regeneration of used materials, electronic dosimeters can be recycled countless times. In this case, it is important to maintain and recalibrate the integrated sensors regularly to ensure correct measurement. Some compromises may have to be made in terms of reproducibility and the validity of the calibration.

An important fact that also has to be considered when choosing a suitable dosimeter type is the sampling rate, in contrast to temporal resolution. Whereas chemical and biological dosimeters continuously integrate incident radiation, electronic dosimeters perform discontinuous measurements at regular time intervals. The larger these intervals are specified, the larger the uncertainties [39]. It has to be taken into account that measurement intervals are defined as short as possible to minimize this effect. In this context, the available memory space from electronic dosimeters also has to be considered, especially for long-term measurements.

With respect to different measurement requirements, the measurement range is also of great importance. Biological spore dosimeters exist in several versions, varying in sensitivity and measurement range (up to 360 SED at max). Especially if the expected dose is not known beforehand, it is hard to choose the right dosimeter version. Alternatively, a pilot study with different dosimeter versions has to be performed to determine the approximate order of magnitude. It is a similar case with PSF dosimeters. There is only one type of PSF dosimeter with a maximum of 300 SED in existence [11]. It has to be stated that long-term measurements (over several weeks) are only possible in a follow-up with several additional dosimeters. Differences in the size and weight of the different dosimeter types can also be significant when choosing the most suitable system.

Table 3 shows the corresponding properties of the dosimeters based on our findings.

It is also important to note that different parameters have to be known for the correct evaluation of the three different dosimeter types. For X2012-10, the rough geographical location has to be known. In addition, the time of day and date are needed; however, these data are logged within the dosimeter. These data are needed to correct for the change of the solar spectrum throughout the day (so that the solar zenith angle is calculated for every measurement). For the PSF dosimeters, the rough geographical location and the month of the year have to be known, which are also used for correction calculations. For VioSpor dosimeters, the date, time of exposure, temperature, and nature of any rough weather conditions have to be known. Here, most parameters have to be logged externally. Whereas electronic dosimeters can be evaluated independently with the software tools provided, thus ensuring transparent evaluation with the possibility to identify systematic errors immediately, chemical and biological dosimeters have to be evaluated by the manufacturer.

Another advantage of the electronic dosimeters is their expandability through additional features. For example, the UV data logger dosimeter X2012-10 has an acceleration sensor, which is used for troubleshooting. Because of the accelerometer and the time resolution of the data, time periods without any movement—e.g., where the test subject did not wear the dosimeter, as well as periods of detector malfunction—can be identified and discarded. In addition, a variation in a specific dosimeter can be identified, evaluated in the laboratory, and then be corrected for. Since the other dosimeter types are single-use only, it is nearly impossible to identify defects in a single unit that can lead to measurement uncertainties. Last but not least, the possibility to upgrade electronic dosimeters with further sensors—e.g., for measuring temperature, acceleration or the geomagnetic fields—presents an opportunity for various other research applications. In the context of our measurements, several additional advantages of electronic dosimeters could clearly be seen. With a view to the standard deviation of different dosimeter types throughout the measurements, electronic dosimeters constantly showed small values. In combination with the measurements using the MAWS201 weather station and the comparison to DWD measurements, electronic dosimeters appear to provide more reliable results than other dosimeter types and are more traceable to the standards.

For the practical approach to checking the intercalibration of a static weather station and person-worn dosimeters [37], we chose electronic dosimeters. As expected, dosimeters worn on a certain part of the body acquire only a fraction of the incident global radiation. As shown in Figure 3, this proportion can vary greatly from person to person and is essentially determined by personal behavior [31]. However, even with regard to the most highly exposed in this example, the proportion does not reach 50% of total global irradiation, but instead is below 15% on average. Extreme values occur when people seek the sun directly to tan or enjoy the warmth. Situations in queues that do not allow for shade seeking also contribute to high exposure levels. The last case in particular, compulsion, comes close to occupational conditions. High daytime exposures are also to be expected here, as exposure is dictated by working conditions. The lack of preventive measures has an aggravating effect here. In the present case, the person with the highest exposure in particular was, according to her own statement, a “sun worshipper” with a desire to tan. This study was a locally limited project whose conclusions still need to be consolidated in follow-up studies.

The presented study faces certain limitations. Strictly speaking, the conclusions drawn are only valid for the dosimeter types at hand. Similarly, working units from other manufacturers may behave differently, but calibration against national (e.g., in Germany: DIN 5031-11 [40]) or international standards should prevent this. For the future, it would be of great benefit if other dosimeter types could be included in such intercomparisons.

The comparison of the statically determined exposure values to the exposure values determined on the person still needs to be validated and, if necessary, detailed by evaluating larger and activity-related groups of test persons.

## 5. Conclusions

In the future, it will be decisive whether measured values from measuring stations can be used in prevention, i.e., for the direct protection of people. If we look at the literature, we can ultimately identify two approaches to the measurement of UV-related exposures in prevention strategies. Studies published so far with various measuring devices focus on the determination of exposure values. Various types of dosimeters have been used in both occupational and private settings [43,44]. Other studies chose the path via the UV index, which is determined via static measurements (ground-based/satellite-based) [45,46]. Here, only general statements can be made about the possible maximum exposure, but this information is useful in the design of apps, for example. It is not yet common practice to link the two worlds. This work is also intended to serve this purpose. A classification is therefore difficult and can only be compared with modelling [47,48,49].

In current research, urban planning plays a key role in addressing the adverse effects of climate change. In many studies addressing this topic, the determination of personal UV exposure is the basis for modelling and urban planning strategies in order to create healthy UVR environments [50,51]. The simultaneous measurement of UV exposure on a stationary surface and on the person makes a decisive contribution to the transferability of values measured at measuring stations to those measured on people [45,52]. Therefore, it was important in this work to check the intercalibration of a static weather station and the person-worn dosimeters.

Many working groups have collected valuable data over the decades to provide an internationally consistent view of UV exposures [43,44]. Efforts in recent years to generate further information from this, such as the determination of disease burdens or dose-response relationships, have often run aground because of the incomparability of studies. If a technical comparability of dosimeters were available or could be derived, this would represent a significant first step.

Since there are several studies in existence, each of which usually deals with only one of the dosimeter types investigated [44], as yet there are none that allow direct intercalibration of different measurement principles. With our study, we have taken a step to close this gap. Nevertheless, there are additional factors influencing personal dosimetric measurements, such as the position of the dosimeters or the latitude (which influences the incident angle of solar radiation), that will need to be considered and further investigated.

In the future, it seems important that such a comparison as presented in this paper be extended to include further analysis of the allocation of dosimeter types to different body sites on the person or a mannequin [53]. This work is underway in some places and will also lead to a high degree of comparability in international studies. Further research on this topic could address the influence of different weather conditions. Measurements of this kind should not only result in the daily indication of a UV index, but can also be useful for the selection of personal protective measures. For this purpose, a specification of individual occupations is desirable, especially in the occupational area. Data of this kind is currently being evaluated [54]. Such information on the actual exposure of a person can be incorporated into a logistical prevention concept to maximize the compliance of tailored protective measures. This work is intended to better compare studies with different measurement approaches and thus enable the construction of international exposure registries and job exposure matrices (JEM) [4,55]. The goal must be to reduce UV-induced skin diseases worldwide and to show people in general how to deal with solar radiation in a healthy way. The professional environment will be strongly affected and benefit from this.

## Figures and Tables

**Table 1:** Measurement parameters for determined measurement scenarios. Incidence of 0° refers to parallelity to the surface normal of the detector, while all other angles also refer to the surface normal. At 110°, the sun does not shine directly onto the dosimeters. Measurement times refer to Central European Summer Time (CEST).

Measurement Number	Date	Time [CEST]	Incident Angle of Solar Radiation	Weather Conditions
1	26 May 2020	07:00–17:00	0°	Sunny
2	29 May 2020	07:00–17:00	30°	Sunny
3	18 June 2020	08:00–17:00	0°	Partly cloudy
4	19 June 2020	12:00–15:00	0°	Partly cloudy
5	23 June 2020	07:00–10:00	0°	Sunny
6	24 June 2020	07:00–17:00	110°	Sunny
7	26 June 2020	07:00–17:00	60°	Sunny
8	10 September 2020	08:00–17:00	85°	Sunny

**Table 2:** Mean UV doses and corresponding standard deviations (in brackets) for each measurement expressed in J/m^2^.

Measurement Number	1	2	3	4	5	6	7	8
Time [CEST]	07:00–17:00	07:00–17:00	08:00–17:00	12:00–15:00	07:00–10:00	07:00–17:00	07:00–17:00	18:00–17:00
Incident Angle of Solar Radiation	0°	30°	0°	0°	0°	110°	60°	85°
	**Mean UV Dose (Standard Deviation) [J/m^2^]**							
X2012-10 V1	4010 (160)	3590 (170)	2540 (110)	1190 (60)	461 (17)	1100 (60)	3210 (190)	1300 (60)
X2012-10 V3	3690 (85)	3220 (160)	2310 (70)	1090 (36)	408 (9)	1001 (48)	2870 (140)	1150 (36)
PSF:	5390 (380)	4550 (900)	3280 (260)	1400 (160)	520 (100)	1200 (180)	3810 (490)	870 (50)
PSF + Filter:	5520 (310)	4150 (190)	3410 (200)	1450 (70)	560 (60)	880 (70)	2210 (190)	747 (8)
VioSpor type III	4970 (30)	3190 (40)	3210 (50)	1190 (30)	1161 (17)	2430 (700)	5290 (30)	810 (740) ^1^
VioSpor type II	3990 (130)	3200 (6)	2923 (210)	1196 (4)	-	-	-	-

^1^ One of the dosimeters was below the evaluation threshold of 150 J/m^2^. In this case, only the other four dosimeters were included in the evaluation.

**Table 3:** Assessment of different properties for the three dosimeter types (electronic, PSF, biological), ranging from very good (+++) to neutral (o) to very poor (−−−).

Property	X2012-10	PSF	VioSpor
Price (short term)	−−−	+++	++
Price (long term)	++	o	−−
Temporal resolution	+++	−−−	−−−
Reproducibility	++	+	−
Sampling rate	o	++	++
Measurement range	+++	−	−−−
Size and weight	o	+++	++
Ease of use (for subject)	+	++	++
Evaluation process	+++	−	−
Data validity	+++	+	+
Upgradability	+++	−−−	−−−

## Data Availability

Data is contained within the article.
